# Impact of Copolymer Architecture on Demicellization and Cargo Release via Head-to-Tail Depolymerization of Hydrophobic Blocks or Branches

**DOI:** 10.3390/polym16081127

**Published:** 2024-04-17

**Authors:** Christos Gioldasis, Apostolos Gkamas, Costas Vlahos

**Affiliations:** Chemistry Department, University of Ioannina, 45110 Ioannina, Greece; c.gioldasis@uoi.gr (C.G.); gkamas@uoi.gr (A.G.)

**Keywords:** molecular dynamics, self-assembly, depolymerization, demicellization, cargo release

## Abstract

Utilizing molecular dynamics simulations, we explored the demicellization and cargo release dynamics of linear and miktoarm copolymers, featuring one, two, and three hydrophobic blocks or branches, each capable of head-to-tail depolymerization. Our findings revealed that, under stoichiometric trigger molecule concentrations, miktoarms with three branches exhibited consistently faster depolymerization rates than those with two branches and linear copolymers. Conversely, at constant trigger molecule concentrations, the depolymerization rates of copolymers exhibited more complex behaviors influenced by two opposing factors: the excess of trigger molecules, which increased with a decrease in the number of hydrophobic branches or blocks, and simultaneous head-to-tail depolymerization, which intensified with an increasing number of branches. Our study elucidates the intricate interplay between copolymer architecture, trigger molecule concentrations, and depolymerization dynamics, providing valuable insights for the rational design of amphiphilic copolymers with tunable demicellization and cargo release properties.

## 1. Introduction 

Hydrophobic drugs and nucleic acid vectors used in therapeutics face significant challenges, including low stability, in vivo degradation, and difficulties in reaching the target site [1]. Consequently, a robust delivery system is essential to transporting these agents safely and efficiently. In recent years, polymeric micelles have emerged as highly effective cargo carriers, owing to their biocompatibility, facile preparation, tunable size and shape, prolonged stability in blood circulation, and enhanced in vivo retention at the target site [2,3,4,5,6]. Typically, polymeric micelles consist of pre-synthesized diblock copolymer chains, comprising a hydrophilic and a hydrophobic block, or a mixture of double hydrophilic diblock copolymers with neutral and oppositely charged blocks, that become hydrophobic upon complexation [7,8]. The hydrophobic segments form the micelle core, while the hydrophilic ones create the surrounding corona. These micelles are derived from dilute polymer concentrations exceeding the critical micelle concentration (CMC). Recently, innovative assembly models (PISA and PIESA) have allowed micellization at high monomer concentrations, combining diblock copolymer synthesis and micellization in a single process [9,10]. The coassembly of cargo molecules with copolymer chains results in the encapsulation of hydrophobic cargo within the micelle core due to the hydrophobic interactions with the solvent. 

Smart polymeric micelles, responsive to the microenvironment of the target site, can be designed by incorporating stimuli-responsive switches, either assembled into micelles or covalently grafted onto copolymer chains. These switches, responsive to pH, light, heat, redox reactions, and enzymes, facilitate efficient drug delivery [11,12,13]. However, challenges may arise in achieving complete cargo release due to the incomplete dissociation of the micelle. A novel and appealing idea for crafting materials with dual characteristics of degradability and responsiveness to stimuli involves end-capped cascade degradable polymers [14,15]. In this approach, polymers consist of a stable backbone while the end-cap remains intact. However, once the end-cap is removed through a specific bond cleavage, a functional group becomes exposed at the polymer’s end. This exposed functionality triggers a series of intramolecular reactions, ultimately leading to the complete depolymerization of the material from end to end. Originally introduced in dendritic systems, this concept showcased the degradation process via an intramolecular cascade, liberating multiple molecules from the periphery of the dendrimer [16]. These systems were subsequently refined to enable the simultaneous release of various drug molecules, the incorporation of targeting elements for tumors, and the inclusion of focal point groups sensitive to either reducing conditions or enzymes. Polymeric micelle carriers with degradable copolymer chains have gained attention for offering controlled yet complete release of cargo molecules and preventing the accumulation of micelles at the target point [17,18,19].

In a noteworthy study, Kim et. al. designed amphiphiles based on poly(benzyl-ether) capable of selective demicellization through head-to-tail depolymerization triggered by hydrophobic fluolid molecules [20]. A labile end-capping unit attached to the end of the hydrophobic block is buried inside the core of the micelle, preventing detachment of the labile units and rendering the demicellization signal specific. Fluolid molecules trigger the detachment of the end-capping unit in the hydrophobic block, after which the entire chain spontaneously and continuously depolymerizes in a head-to-tail manner without the need for additional stimuli. Loading doxorubicin inside the micelles led to their molecular-level degradation, resulting in the controlled and complete release of the cargo molecules.

The depolymerization-induced disassembly mechanism has primarily been explored for amphiphiles with a linear chain architecture carrying one labile end-cap unit. The design of amphiphiles with a different architecture, bearing more labile end-cap units, can further enhance the control of depolymerization and cargo release, which is useful for biological and environmental applications. Due to the lack of such studies, we conducted coarse-grained molecular dynamics simulations of micelles formed by (a) linear AB diblock copolymer with one hydrophilic (A–type) and one hydrophobic (B–type) block, featuring one end-cap unit; (b) miktoarm star copolymer A(B)_2_ with one hydrophilic and two hydrophobic branches, equipped with two end-cap units; and (c) miktoarm star copolymer A(B)_3_ with three hydrophobic branches and three end-cap units. We first provide a brief introduction to the coarse-grained model and the interaction parameters used for simulating amphiphilic copolymers under selective solvents. Additionally, we outline the algorithm for depolymerization reactions and describe the Python algorithm employed to study micellization, loading capacity, and cargo release. In the results section, we present calculations of the mass distributions of micelles formed by different copolymers both before and after cargo loading. Furthermore, we analyze the kinetics of head-to-tail depolymerization of hydrophobic blocks or branches, as well as the kinetics of cargo release triggered by hydrophobic small molecules. Finally, we summarize our findings briefly and offer some concluding remarks.

## 2. Model

### 2.1. Coarse-Grained Molecular Dynamics Simulation Details

**Figure 1:**
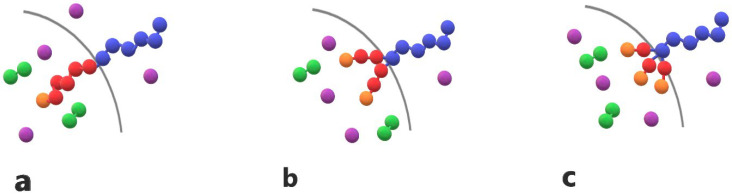
Cartoon representations of (**a**) linear diblock copolymer A_6_B_6_, (**b**) miktoarm star copolymer A_6_(B_3_)_2_, and (**c**) miktoarm star copolymer A_6_(B_2_)_3_ with one, two, and three end-cap beads (orange) are presented. T–type trigger molecules (purple) and cargo molecules (green) C_2_ are also depicted. Hydrophilic A–type beads and hydrophobic B–type beads are shown in blue and red, respectively.

Amphiphilic AB linear diblock copolymers, miktoarm star copolymers, and cargo molecules are modeled as bead-spring chains consisting of Lennard–Jones beads with a diameter σ following the Murat–Grest model [21]. In all simulations, the copolymer chains collectively consist of 30 A–type hydrophilic and 30 B–type hydrophobic beads. In linear AB diblock copolymers, denoted as A_30_B_30_, the hydrophilic and hydrophobic beads are distributed in one block each. In miktoarm star copolymers with three and four branches, denoted as A_30_(B_15_)_2_ and A_30_(B_10_)_3_, the thirty hydrophobic beads are distributed into two or three branches, respectively. The free end bead of the hydrophobic block or branch is designated as an end-cap unit. The cargo molecules are linear chains containing three C–type hydrophobic beads, denoted as C_3_. The trigger molecule consists of a single T–type hydrophobic bead with a diameter σ. All copolymer chains, including those with linear and star architecture, as well as cargo and trigger molecules, are illustrated in Figure 1. 

In the current study, simulations were contacted for mixtures comprising (a) A_30_B_30_ with C_3_, (b) A_30_(B_15_)_2_ with C_3_, and (c) A_30_(B_10_)_3_ with C_3_, collectively containing 1000 copolymer chains and 2000 or 4000 cargo molecules. In all cases, the total concentration of beads (including copolymer and cargo beads) was maintained at [*Φ*] = 0.12 where most micelles are formed. 

Excluded volume bead-bead interactions were considered to mimic the macroscopic solvent conditions. These interactions were calculated using a truncated Lennard–Jones potential [8,22]:

(1) $$U_{LJ}\left(r_{ij}\right)=\left\{\begin{array}{c} 4\varepsilon \left[{\left(\frac{\sigma }{r_{ij}}\right)}^{12}-{\left(\frac{\sigma }{r_{ij}}\right)}^{6}-{\left(\frac{\sigma }{r_{cij}}\right)}^{12}+{\left(\frac{\sigma }{r_{cij}}\right)}^{6}\right],\;r_{ij}\leq r_{cij}\\ 0,r_{ij}>r_{cij}\; \end{array}\right.$$

where *ε* is the well depth and *r*_cij_ is the cutoff radius. Different beads were connected with finitely extensible nonlinear elastic bonds (FENE). The FENE potential is expressed as [8]

(2) $$U_{Bond}\left(r_{ij}\right)=\left\{\begin{array}{c} -0.5kR_{0}^{2}ln\left[1-{\left(\frac{r_{ij}}{R_{0}}\right)}^{2}\right],\;\;r_{ij}\leq R_{0}\\ \infty ,{\;\;\;\;\;\;\;\;\;\;\;\;\;\;\;\;\;\;\;\;\;\;\;\;\;\;\;\;\;\;\;\;\;\;\;\;\;\;\;\;\;\;\;r}_{ij}>R_{0}\; \end{array}\right.$$

where *r*_ij_ is the distance between beads *i* and *j*, $k=25\varepsilon /\sigma^{2}$ and *R*_0_ is the maximum extension of the bond [21] ($R_{0}=1.5\sigma )$. The solvent molecules are implicitly considered. 

Molecular dynamics simulations with a Langevin thermostat were conducted in a cubic box with periodic boundary conditions, using the open-source massive parallel simulator LAMMPS [23]. The reduced temperature of the simulation T* was set to T* = *k*_B_*T/ε* = 1.8 (*ε* = 1) corresponding to bad solvent conditions [8]. Different cutoff distances and epsilon parameters in the Lennard–Jones potential were employed to describe the interactions between copolymer units. The B–B, B–C, and C–C interactions were considered attractive. Specifically, the B–B interactions consistently corresponded to T* = 1.8 while the B–C and C-C interactions were even more attractive, corresponding to lower temperatures (T* = 1.8, 1.4, and T* = 1.8, 1.6, 1.5 and 1.4, respectively) with a cutoff distance *r*_cij_ of 2.5*σ* (*ε*_BB_ = 1, *ε*_ij_ = T*/1.8, for *i*, *j* ≠ B). Conversely, the A–A, A–B, and A–C interactions were considered repulsive, with a cutoff distance *r*_cij_ of 2^1/6^σ. In the latter case, the Lennard–Jones potential is shifted by *ε*. For simplicity, all types of beads were assumed to have the same mass (m *=* m_i_ =1) and diameter (σ = 1).

Following the Stillinger criterion [24], two chains were considered to reside in the same micelle if the distance between any two nonbonded hydrophobic beads belonging to different chains was found to be within 1.5σ.

We performed 2 million timesteps with an integration step t=0.006τ ($\tau =\sqrt{\frac{{m\sigma }^{2}}{\varepsilon }})$, setting all cutoff radii equal to *r*_cij_ *=* 2^1/6^σ to eliminate any bias introduced from the initial conformation. Subsequently, the system was allowed to equilibrate for 30 million timesteps. The simulation was then extended to 300 to 600 million timesteps. The duration of the simulation was evaluated by calculating the tracer autocorrelation function [7]:

(3) $$C\left(t\right)=\frac{\langle N\left(t_{0}+t\right)N(t_{0})\rangle -{\langle N(t_{0})\rangle }^{2}}{\langle N^{2}\left(t_{0}\right)\rangle -{\langle N(t_{0})\rangle }^{2}}\;\;\;\;,$$

where *N*(*t*) is the number of molecules in the micelle in which the copolymer resides at time *t*. We considered all copolymers as tracers, with each time step serving as the time origin *t*_0_. The characteristic relaxation time *t*_relax_ is defined as the time required for *C*(*t*) to reach the value [7,8] of 1/e = 0.37. Each simulation was conducted for a minimum of 10*t*_relax_ to ensure 10 independent conformations. The properties of interest were calculated as averages from 4000 snapshots using the block average method with ten blocks. 

### 2.2. Depolymerization and Cluster Analysis

**Figure 2:**
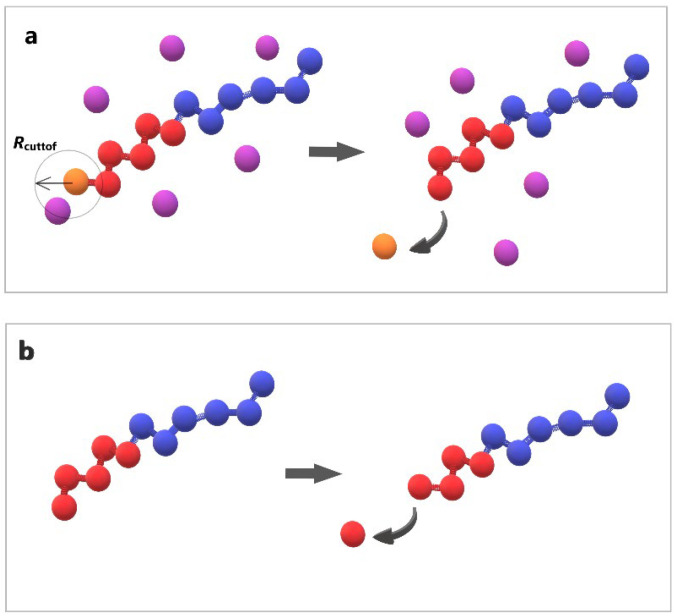
Cartoon representation of the depolymerization algorithm: (**a**) the breaking of a bond between the end-cap (orange) and B–type bead (red) triggered by a T–type molecule (purple); (**b**) the head-to-tail breaking of a bond between two B–type beads (red) without stimuli. Hydrophilic A–type beads are shown in blue.

The stochastic depolymerization of the hydrophobic monomers of amphiphilic copolymers, as depicted in Figure 2, utilized the “bond/react” functionality of LAMMPS. This feature allows the breaking of bonds between beads based on distance-dependent probabilistic criteria. Topology changes are specified in pre- and post-reaction molecule templates. Two distinct depolymerization steps were undertaken: (a) the breaking of bonds between the hydrophobic end-cap and B–type beads, triggered by the presence of T–type molecule within *R*_cutoff_ = 1σ from the end-cap bead, with a predetermined reaction probability (RP) (Figure 2a); (b) the spontaneous propagation of depolymerization of hydrophobic beads with a predetermined RP occurring without any external stimuli (Figure 2b). RPs are adjustable parameters. 

We utilized graph clustering analysis to analyze the simulation data. Initially, we identified micelles employing the data clustering algorithm DBSCAN implemented in the Python library Sklearn [25], with a maximum allowable neighborhood radius of 1.5σ. For a point (bead) to be classified as a core point, a minimum of two points (including the point itself) must be within the neighborhood. A precomputed neighbor sparse array was utilized as the input for the DBSCAN algorithm. To construct this array, the KDTree neighbor data structure from the Python library SciPy [26] was employed, specifically the Sparse Distance Matrix algorithm with a maximum distance of 1.5σ between two points. It is worth noting that the distance matrix algorithm disregards points with a distance greater than the maximum distance parameter. The KDTree neighbor data structure takes periodic boundary conditions into account, ensuring that the clustering analysis includes the periodic images.

For the identification of polymer chains, we employed the Python NetworkX library [27]. In this context, beads were represented as nodes, and bonds were represented as edges. From the graph created by this library, we extracted the polymer chains using the “Connected/Components” algorithm. This algorithm generates connected components from a graph, corresponding to bead spring chains in our case. Subsequently, the polymer chains were assigned to micelles based on the previous steps. To compute properties such as the radius of gyration of the micelles (core, corona, and total) and the shape anisotropy parameter *κ*^2^, we utilized the outbox coordinates. To achieve this, micelles that split due to the periodic conditions (inbox coordinates) were identified and consolidated using the data clustering algorithm DBSCAN, the KDTree neighbor data structure, and the Sparse Distance Matrix algorithm without periodic conditions. Our testing, employing 250, 500, and 750 copolymer chains, revealed varying aggregation numbers of micelles formed by linear copolymers with identical interaction energy parameters. However, with 1000 and 1250 copolymer chains, consistent results were consistently achieved, indicating that 1000 chains represent the threshold where finite size effects dissipate. Consequently, employing all-atom simulations for such large systems is prohibited.

## 3. Results and Discussion

### 3.1. Micellization and Cargo Encapsulation

**Figure 3:**
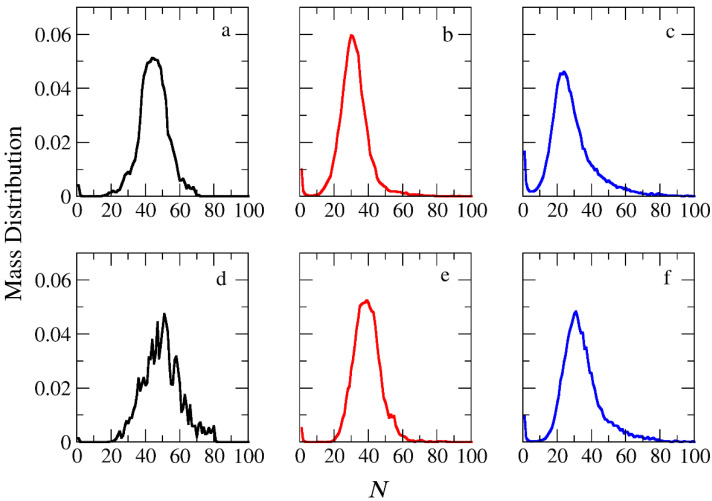
Mass distribution of micelles as a function of the aggregation number *N* formed by (**a**) A_30_B_30_, (**b**) A_30_(B_15_)_2_, and (**c**) A_30_(B_10_)_3_ copolymers. ibid. for mixtures: (**d**) A_30_B_30_ and C_3_, (**e**) A_30_(B_15_)_2_ and C_3_, and (**f**) A_30_(B_10_)_3_ and C_3_. In all simulations, [*Φ*] = 0.12.

The micellization of amphiphilic linear A_30_B_30_, miktoarm star A_30_(B_15_)_2_, and A_30_(B_10_)_3_ copolymers was studied prior to cargo encapsulation at T* = 1.8 and [*Φ*] = 0.12. We calculated the aggregate mass distribution, the gyration radius, and the shape anisotropy of micelles. In Figure 3, the mass distribution of the aggregates is depicted. Peak values corresponding to the preferential aggregation number *N*_p_ are higher for linear copolymers and decrease as the number of arms in miktoarm copolymers increases, aligning with previous simulation results [28,29]. 

The encapsulation of cargo molecules was examined for different interaction parameters between hydrophobic C–C and B–C beads. Simulations of mixtures containing 1000 copolymer chains with 2000 or 4000 cargo molecules C_3_ were contacted at [*Φ*] = 0.12, and the results are presented in Table S1. For linear A_30_B_30_ copolymers, an increase in the strength of attractions between C–C beads from T*_C–C_ =1.8 to 1.4 had a minimal effect on the percentage of encapsulated cargo molecules (41% to 43%). In contrast, a similar increase in the strength of attractions between B–C beads (T*_B–B_ = 1.8 and T* _B–C_ = T*_C–C_ = 1.4) dramatically increased the percentage of encapsulated molecules to 83%, aligning with previous findings [30]. The copolymer architecture did not influence the encapsulation of cargo molecules, as the driving force for this process is the hydrophobicity between B and C beads. In a mixture of A_30_B_30_ with 4000 C_3_ molecules and attractive interactions corresponding to T*_B–B_ = 1.8 and T*_B–C_ = T*_C–C_ = 1.4, the percentage of encapsulated cargo molecules remained constant at 82%. Free cargo molecules were in dynamic equilibrium with the encapsulated ones. The loading capacity [20] (LC) of the micelle, defined as the *w*/*w* percentage of the cargo accommodated in the loaded micelle carrier for the two different cargo concentrations, was found to be 7.6% and 14.1%, respectively. The encapsulation of hydrophobic molecules in the micelle core altered the hydrophilic/hydrophobic balance, triggering the association of free copolymer chains into the micelles and resulting in a higher aggregation number for all copolymer architectures (Figure 3 and Table S2). The *N*_p_ of loaded micelles with LC = 7.6% increased by approximately 16%, 30%, and 35% for the linear A_30_B_30_, miktoarm star A_30_(B_15_)_2_, and A_30_(B_10_)_3_ copolymer mixtures, respectively. This higher increase in *N*_p_ in miktoarm copolymers reflects the more efficient packing of B and C beads in the micelle core, facilitating the decrease in free energy. The autocorrelation function indicates that the micelles of A_30_B_30_ copolymers with LC = 14.1% were kinetically frozen even after 600 million timesteps and were not further studied. 

**Figure 4:**
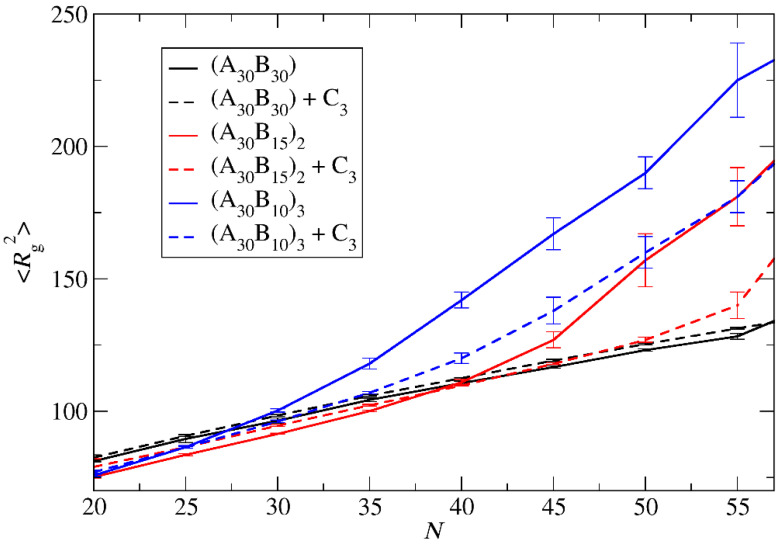
The mean squared radius of gyration for ‘empty’ micelles formed by A_30_B_30_, A_30_(B_15_)_2_, and A_30_(B_10_)_3_ copolymers (solid lines), and for loaded micelles formed by mixtures with 2000 C_3_ cargo molecules (dashed lines) plotted against *N*. Standard deviation is indicated by the error bars.

**Figure 5:**
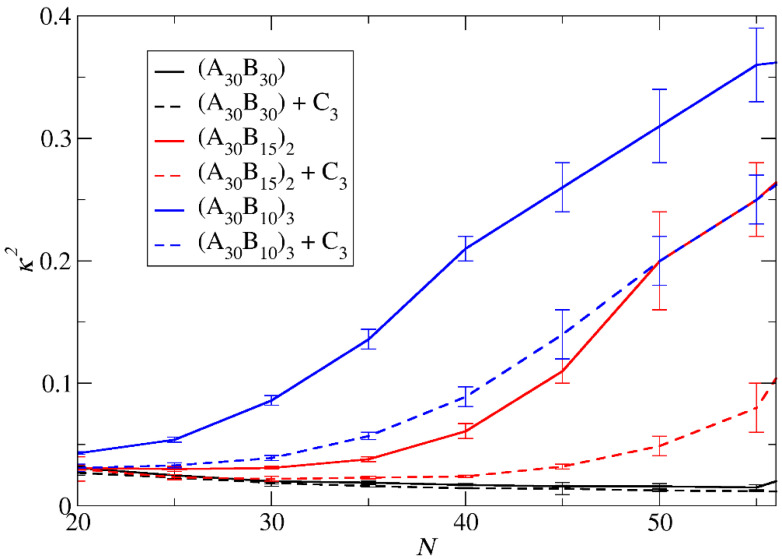
The mean shape anisotropy parameter *κ*^2^ for ‘empty’ micelles formed by A_30_B_30_, A_30_(B_15_)_2_, and A_30_(B_10_)_3_ copolymers (solid lines), and for loaded micelles formed by mixtures with 2000 C_3_ cargo molecules (dashed lines) plotted against *N*. Standard deviation is indicated by the error bars.

The mean squared radius of gyration <*R*_g_^2^>, serves as a measure expressing the size of both ‘empty’ and loaded micelles. The calculation of <*R*_g_^2^> focused solely on copolymer beads to maintain consistent molecular weight in micelles sharing the same aggregation number *N*. In the case of preferential micelles, <*R*_g_^2^> was observed to be higher in loaded micelles by approximately 9%, 16%, and 16% for A_30_B_30_, A_30_(B_15_)_2_, and A_30_(B_10_)_3_ copolymers, respectively. This elevation can be attributed to the increased *N*_p_ of loaded micelles, with the disparity in *N*_p_ becoming more pronounced in miktoarm star copolymers. Figure 4 illustrates the results for <*R*_g_^2^> for both types of micelles with the same *N*_p_. For A_30_B_30_ copolymers, <*R*_g_^2^> is marginally higher in loaded micelles, a consequence of cargo molecules enlarging the micelle core and leading to a slight increase in <*R*_g_^2^>. A similar trend is observed in micelles formed by A_30_(B_15_)_2_ and A_30_(B_10_)_3_ copolymers up to *N* = 40 and 27, respectively. However, for higher *N* values, the <*R*_g_^2^> of ‘empty’ micelles surpasses that of loaded ones. The anisotropy shape parameter [29,31] *κ*^2^ values depicted in Figure 5 reveal that ‘empty’ micelles exhibit an elongated shape in contrast to the more spherical-shaped loaded micelles. This distinction arises from the improved packing of hydrophobic content in the micelle core, leading to higher <*R*_g_^2^> values for ‘empty’ micelles compared to the loaded ones. Notably, A_30_B_30_ copolymers form very spherical micelles in both ‘empty’ and loaded states, while the differences in *κ*^2^ values for A_30_(B_15_)_2_ and A_30_(B_10_)_3_ copolymers increase with the number of hydrophobic arms.

### 3.2. Kinetics of Depolymerization

The depolymerization and subsequent degradation of loaded micelles were carried out using two distinct concentrations of hydrophobic trigger molecules. The first concentration aligns with the stoichiometry of the total end-cap beads in A_30_B_30_, A_30_(B_15_)_2_, and A_30_(B_10_)_3_ copolymer solutions, accounting for 1000, 2000, and 3000 T–type molecules, respectively. The second concentration is maintained at a constant level, with 4000 T–type molecules applied to all copolymer solutions. These trigger molecules were randomly inserted into the simulation box, representing the final snapshot of the trajectory of copolymer comicellization involving cargo C_3_ molecules. This configuration served as the initial state for the depolymerization process. The introduction of trigger molecules led to an adjustment in overall solution concentration, reaching up to [*Φ*] = 12.6. The attractive interactions between T–T, B–T, and T–C beads consistently corresponded to T* = 1.8, while the T–A interactions were considered repulsive. 

When T–type molecules infiltrate the hydrophobic core of the micelle, they can initiate the rapture of bonds between the hydrophobic end-cap and B–type beads if the distance of T–type molecules is within *R*_cutoff_ = 1σ from the end-cap bead, governed by a predetermined reaction probability, RP_T_. Subsequently, spontaneous head-to-tail depolymerization of hydrophobic B–type beads may occur, guided by a predetermined reaction probability, RP_B_, and this process takes place without any external stimuli. Simulations were conducted to explore scenarios where the end-cap bond-breaking occurs more easily, equivalently, or more difficultly than the B–B bond (with RP_B_ values of 10^−3^, 10^−4^, and RP_T_ values of 10^−2^, 10^−3^, and 10^−4^). 

**Figure 6:**
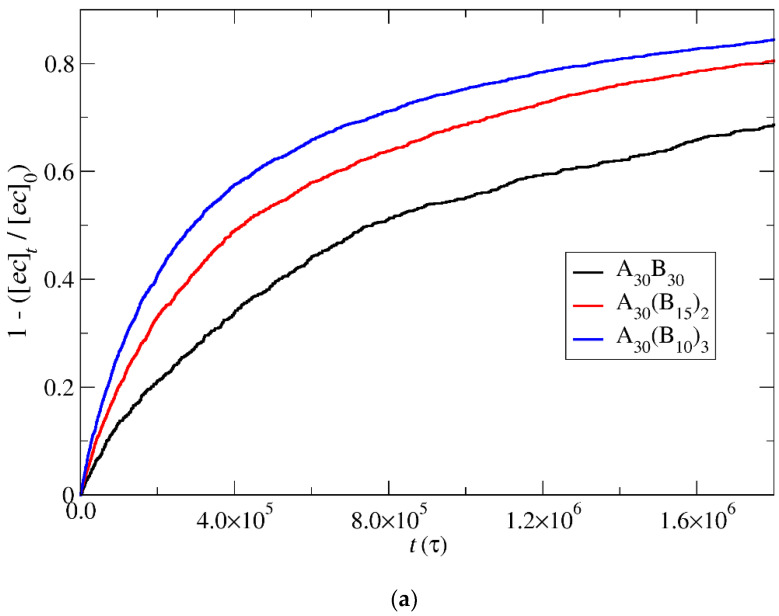

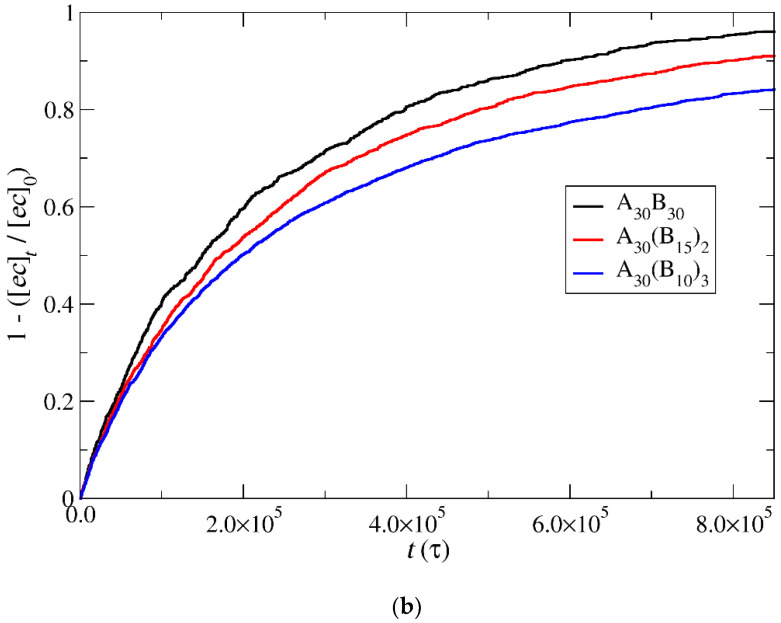
Depolymerization fraction of end-cap beads of A_30_B_30_, A_30_(B_15_)_2_, and A_30_(B_10_)_3_ copolymers as a function of time for (**a**) stoichiometric trigger molecule concentration and (**b**) constant trigger molecule concentration. RP_T_ = 10^−4^, RP_B_ = 10^−3^. [*ec*]_0_ is the initial end-cap beads concentration; [*ec*]_t_ is the end-cap concentration.

The results depicting the fraction of end-cap bonds broken with RP_T_ = 10^−4^ and RP_B_ = 10^−3^ are showcased in Figure 6 for both stoichiometric and constant concentrations of trigger molecules. Notably, the depolymerization of end-cap beads in A_30_(B_10_)_3_ copolymers at a stoichiometric concentration of trigger molecules surpasses the rates observed in A_30_(B_15_)_2_ and A_30_B_30_ copolymers. This heightened efficacy is attributed to the doubled and tripled presence of T–type molecules in A_30_(B_10_)_3_ mixtures compared to their counterparts in A_30_(B_15_)_2_ and A_30_B_30_ mixtures, thereby increasing the probability of infiltration into the hydrophobic core. Furthermore, the elongated sphere scheme (depicted in Figure 5) of A_30_(B_10_)_3_ micelles contribute to a larger core surface exposed to the solvent compared to other copolymers. This structural characteristic facilitates the penetration of trigger molecules, contributing to the observed faster depolymerization in A_30_(B_10_)_3_ copolymers. Conversely, contrasting trends are noted in the depolymerization process under constant T–type molecule concentration (Figure 6b). The excess of trigger molecules is fourfold, twofold, and 1.5 times higher than the stoichiometric case for A_30_B_30_, A_30_(B_15_)_2_, and A_30_(B_10_)_3_ mixtures, respectively, leading to quicker penetration of trigger molecules and depolymerization of linear copolymers followed by miktoarm star copolymers. Various factors, including micelle corrosion, also influence the penetration of trigger molecules into the micelle core and, subsequently, the breaking of the end-cap bonds with B–type beads. Higher values of RP_T_ simply reduce the time for the completion of end-cap removal without affecting the order of copolymer depolymerization in both stoichiometric and constant concentration cases (Figure S1). Similarly, an increase by x times in the T–type molecule concentrations in all copolymer mixtures results in faster end-cap removal without altering the order of copolymer depolymerization (Figure S2).

**Figure 7:**
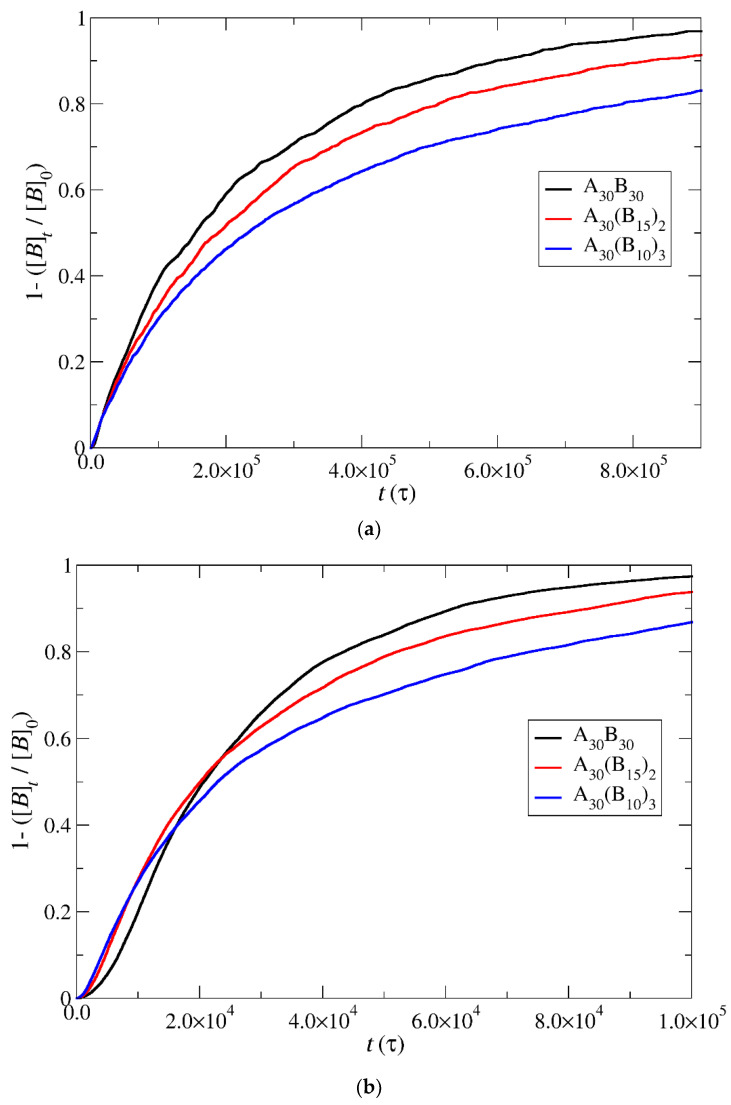

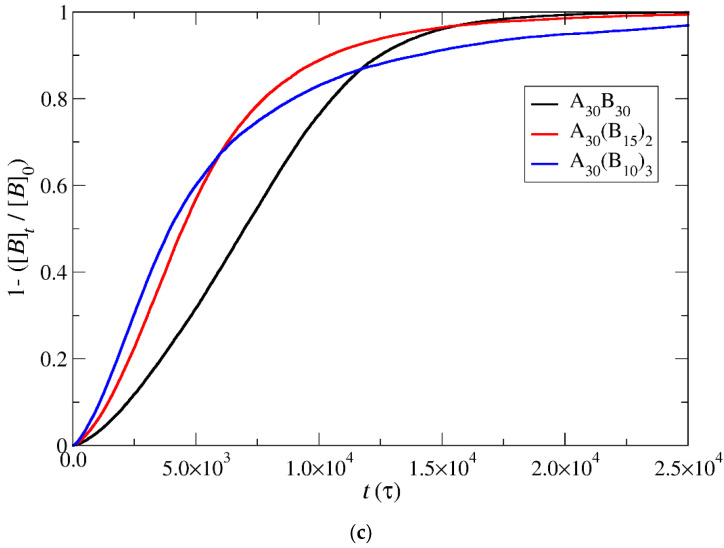
Depolymerization fraction of all hydrophobic beads of A_30_B_30_, A_30_(B_15_)_2_, and A_30_(B_10_)_3_ copolymers plotted against time for constant trigger molecule concentration: (**a**) RP_T_ = 10^−4^ and RP_B_ = 10^−3^, (**b**) RP_T_ = 10^−2^ and RP_B_ = 10^−3^, and (**c**) RP_T_ = 10^−3^ and RP_B_ = 10^−3^. [*B*]_0_ is the initial hydrophobic beads concentration; [*B*]_t_ is the hydrophobic beads concentration.

Under constant and stoichiometric concentrations of trigger molecules, the rate of depolymerization is determined by the slope of the depolymerization curves. When the breaking of the end-cap bond with the B–type bead is more challenging than the bond between B–type beads (RP_T_ = 10^−4^, RP_B_ = 10^−3^), the rate of depolymerization for hydrophobic beads is governed by the slower step. Consequently, the depolymerization trends among copolymer architectures align with those observed in the end-cap bond break. Specifically, A_30_B_30_ exhibits faster depolymerization, followed by A_30_(B_15_)_2_ and A_30_(B_10_)_3_ mixtures (Figure 7a) for the same reasons outlined earlier. However, at RP_T_ = 10^−2^ and RP_B_ = 10^−3^, where the breakage of the bond between B–type beads becomes the slower reaction step, the branching architecture significantly influences the depolymerization process. The head-to-tail depolymerization of B–type beads occurs simultaneously in all branches, resulting in faster depolymerization in A_30_(B_10_)_3_ with three branches, followed by A_30_(B_15_)_2_ and A_30_B_30_ with two and one branches, respectively. Notably, when 65% of the total bonds of hydrophobic beads are broken, the depolymerization rate of A_30_(B_15_)_2_ copolymers surpasses that of A_30_(B_10_)_3_ copolymers (Figure 7b). This is attributed to the higher excess of trigger molecules in A_30_(B_15_)_2_ mixtures, which facilitates the faster removal of end-cap beads from the hydrophobic branch. Similarly, the depolymerization rate of A_30_B_30_ copolymers exceeds that of A_30_(B_10_)_3_ and A_30_(B_15_)_2_ copolymers when the fraction of total bond breakage reaches 0.85 and 0.95, respectively. Beyond these values, the order of depolymerization rates reverts to the pattern observed at RP_T_ = 10^−4^ and RP_B_ = 10^−3^. In actuality, the reversal in the depolymerization order occurs in the cases of RP_T_ = 10^−4^ and RP_B_ = 10^−3^, but the crossovers between the depolymerization rates are confined to a narrow range, specifically between 0.03 and 0.06. When RP_T_ = RP_B_ = 10^−3^, the depolymerization rate of hydrophobic beads for different architectures is determined by two opposite factors: the excess of trigger molecules, which increases as the number of hydrophobic branches or blocks decreases, and the simultaneous head-to-tail depolymerization of beads, which intensifies with an increasing number of branches. As depicted in Figure 7c, the crossover of depolymerization curves occurs within the range of 0.20 to 0.55. Beyond this fraction, A_30_B_30_ exhibits faster depolymerization, followed by A_30_(B_15_)_2_ and A_30_(B_10_)_3_ mixtures.

**Figure 8:**
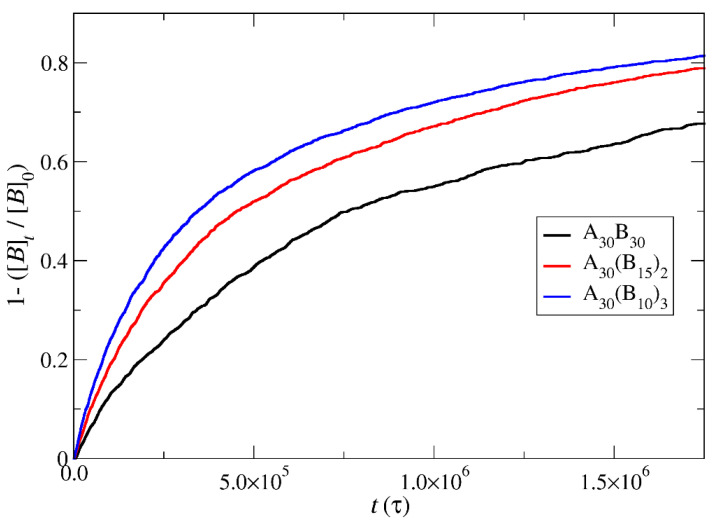
Depolymerization fraction of all hydrophobic beads of A_30_B_30_, A_30_(B_15_)_2_, and A_30_(B_10_)_3_ copolymers plotted against time for stoichiometric trigger molecules concentration. RP_T_ = 10^−4^; RP_B_ = 10^−3^. [*B*]_0_ is the initial hydrophobic beads concentration; [*B*]_t_ is the hydrophobic beads concentration.

In the case of depolymerization of hydrophobic beads under stoichiometric concentration of T–type molecules with the end-cap beads (Figure 8), the rate of depolymerization is consistently faster in A_30_(B_10_)_3_ followed by A_30_(B_15_)_2_ and A_30_B_30_ mixtures. This can be attributed to the same factors explained earlier in the context of end-cap depolymerization, coupled with the higher number of branches that are potentially subject to simultaneous head-to-tail depolymerization of B–type beads. 

### 3.3. Demicellization

**Figure 9:**
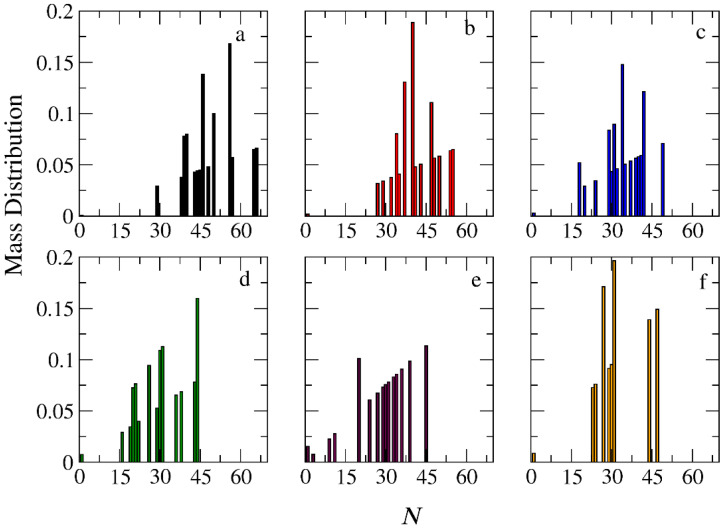
Mass distribution of micelles formed by linear A_30_B_30_ copolymers across various time points and depolymerization fractions of all hydrophobic beads: (**a**) *t* = 0, 0, (**b**) *t* = 135,000τ, 0.15, (**c**) *t* = 360,000τ, 0.31, (**d**) *t* = 630,000τ, 0.45, (**e**) *t* = 1,260,000τ, 0.60, and (**f**) *t* = 1,800,000τ, 0.68. The trigger molecule concentration is maintained stoichiometric for end-cap beads in all cases. RP_T_ = 10^−4^ and RP_B_ = 10^−3.^

**Figure 10:**
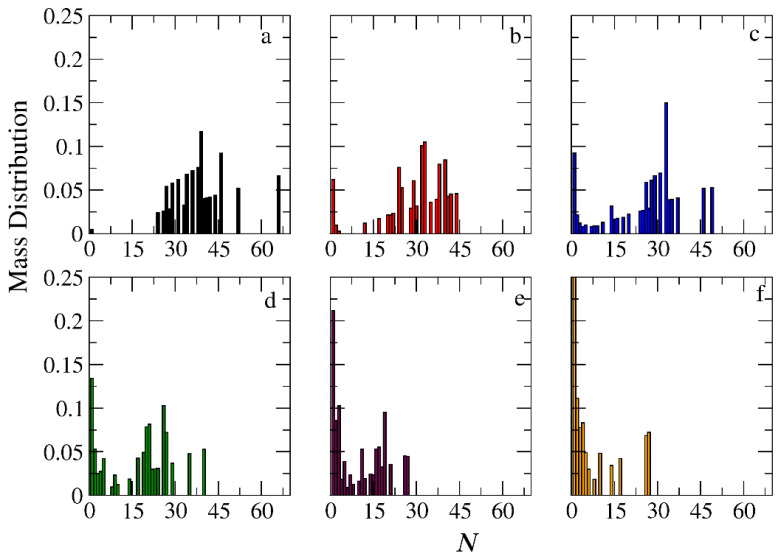
Mass distribution of micelles formed by miktoarm A_30_(B_15_)_2_ copolymers across various time points and depolymerization fractions of all hydrophobic beads: (**a**) *t* = 0, 0, (**b**) *t* = 90,000τ, 0.17, (**c**) *t* = 180,000τ, 0.29, (**d**) *t* = 360,000τ, 0.44, (**e**) *t* = 720,000τ, 0.60, and (**f**) *t* = 1,440,000τ, 0.75. The trigger molecule concentration is maintained stoichiometric for end-cap beads in all cases. RP_T_ = 10^−4^ and RP_B_ = 10^−3^.

**Figure 11:**
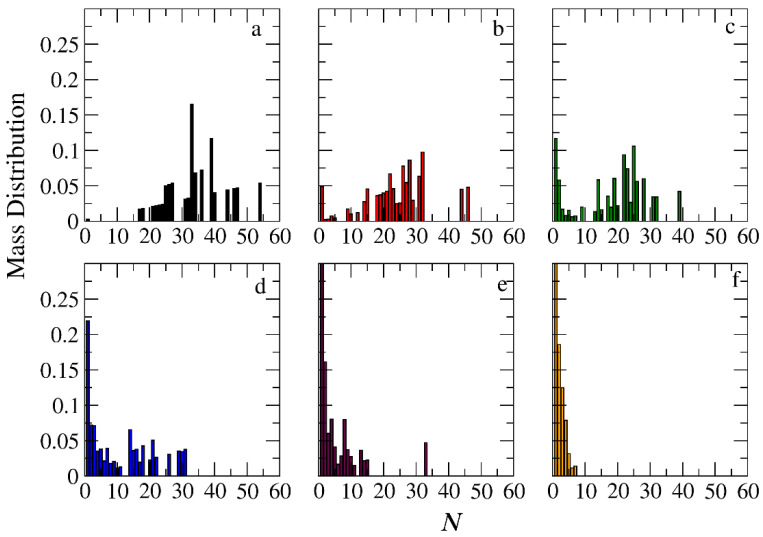
Mass distribution of micelles formed by miktoarm A_30_(B_10_)_3_ copolymers across various time points and depolymerization fractions of all hydrophobic beads: (**a**) *t* = 0, 0, (**b**) *t* = 45,000τ, 0.13, (**c**) *t* = 135,000τ, 0.29, (**d**) *t* = 270,000τ, 0.44, (**e**) *t* = 540,000τ, 0.59, and (**f**) *t* = 1,170,000τ, 0.75. The trigger molecule concentration is maintained stoichiometric for end-cap beads in all cases. RP_T_ = 10^−4^ and RP_B_ = 10^−3^.

The depolymerization of hydrophobic blocks or branches in a head-to-tail fashion results in copolymer chains that are either shorter or lack hydrophobic blocks or branches. The reduction in hydrophobic content within the copolymer corresponds to an increase in the critical micelle concentration. Consequently, these chains migrate into the solution, triggering the gradual demicellization of the mixtures. Figure 9, Figure 10 and Figure 11 and Figures S3–S8 present the results regarding the micelle mass distribution at various fractions of bond breakage between hydrophobic beads (approximately 0, 0.15, 0.30, 0.45, 0.60, and 0.75) for stochiometric and constant concentrations of trigger molecules. Micelle mass distributions were computed from a single snapshot, and their normalization is based on the total micelle content at the specific snapshot. Copolymer chains devoid of B–type beads lose the ability to participate in micelles and are excluded from the count as single chains in the micelle distribution. Across all figures, it is evident that the aggregation number of micelles (*N*) progressively decreases with simulation time. This decline signifies the gradual loss of copolymer chains from micelles without micelle breaking. 

In the case of A_30_B_30_ copolymers with stoichiometric mixtures of trigger molecules and the end-cap beads (RP_T_ = 10^−4^ and RP_B_ = 10^−3^), Figure 9 illustrates that all chains leaving the micelles consist solely of A–type beads. This is attributed to the faster depolymerization of B–type beads in the B–type block of a copolymer chain due to the higher RP_B_ compared to RP_T_. Conversely, in the case of single miktoarm copolymers, the chains exiting the micelles are composed of B–type branches, characterized by a reduced content of B–type beads (Figure 10 and Figure 11). At a bond break fraction of 0.68, the aggregation number of A_30_B_30_ copolymer micelles remains substantial. However, in A_30_(B_15_)_2_ and even more so in A_30_(B_10_)_3_ copolymers, the aggregation numbers diminish at a similar bond break fraction. 

The same trends are observed in the micelle mass distribution for mixtures with a constant concentration of trigger molecules (Figures S3–S5). However, at the same bond break fraction, the aggregation numbers are noticeably smaller. When reaction probabilities for both reactions are equal (RP_T_ = RP_B_ = 10^−3^), micelles and single corroded copolymer chains are in equilibrium, but the aggregation number of micelles is smaller compared to the case with RP_T_ = 10^−4^ and RP_B_ = 10^−3^ at the same constant concentration. Increasing the reaction probability of end-cap bonds (RP_T_ = 10^−2^ and RP_B_ = 10^−3^) leads to even smaller aggregation numbers for micelles of all architectures. Nonetheless, A_30_B_30_ micelles consistently exhibit higher aggregation numbers than A_30_(B_15_)_2_ and A_30_(B_10_)_3_ for the same bond break fraction (Figures S6–S8).

### 3.4. Kinetics of Cargo Release

**Figure 12:**
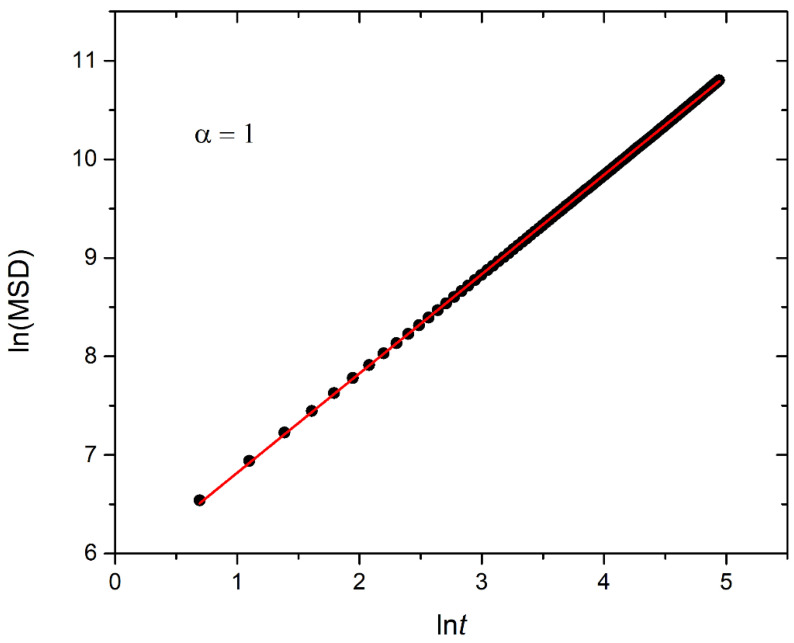
Plot illustrating the mean squared displacement calculated from 90 cargo molecules within an A_30_B_30_ micelle with *N* = 50, against time for stoichiometric trigger molecule concentration with end-cap beads and RP_T_ = 10^−4^ and RP_B_ = 10^−3^. The exponent α corresponds to the scaling behavior of *t*.

**Figure 13:**
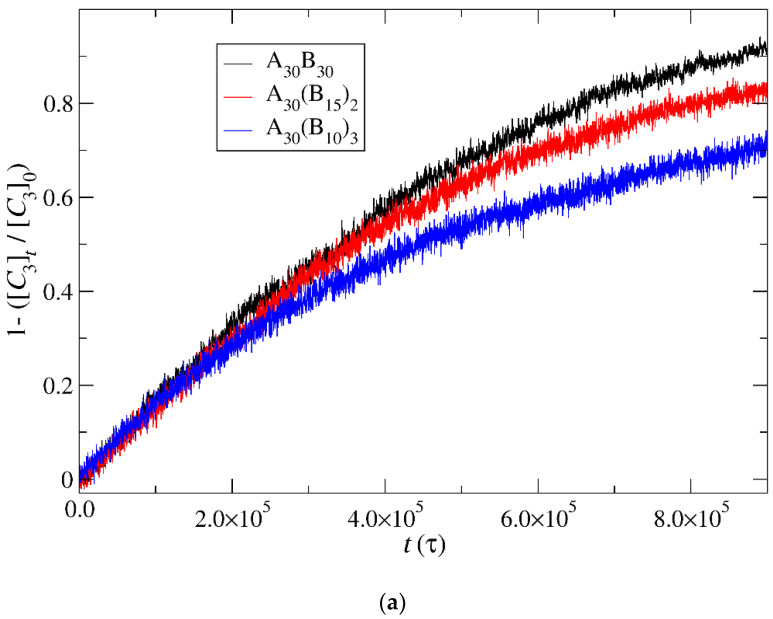

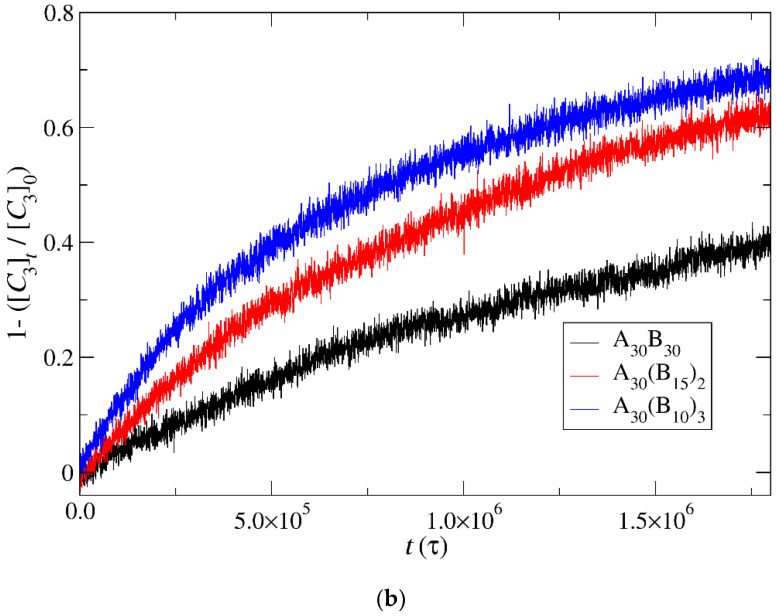
Cargo molecules release fraction from A_30_B_30_, A_30_(B_15_)_2_, and A_30_(B_10_)_3_ copolymer mixtures plotted against time for (**a**) constant trigger molecule concentration and (**b**) stoichiometric trigger molecule concentration with end-cap beads. RP_T_ = 10^−4^ and RP_B_ = 10^−3^. [*C*_3_]_0_ is the initial cargo molecule concentration; [*C*_3_]_t_ is the cargo molecule concentration.

In this section, the mobility of cargo molecules was investigated through the calculation of their mean squared displacement (MSD) as a function of time. The study utilized 90 cargo molecules residing within a micelle formed by A_30_B_30_ copolymers with *N* = 50 for mixtures with RP_T_ = 10^−4^ and RP_B_ = 10^−3^, maintaining a constant concentration of trigger molecules. The results for MSD were averaged across the cargo tracers and are depicted in Figure 12. The exponent α corresponding to the scaling behavior of time was determined to be unity, suggesting normal diffusion in the release of cargo molecules. Notably, even before demicellization occurred in the tracer micelle, a substantial exchange of cargo molecules with different micelles and the solution was observed. Approximately, 18% of tracer molecules left the tracer micelle, while others were inserted, maintaining the number of encapsulation cargo molecules nearly constant. As the micelles underwent depolymerization and corrosion, the number of encapsulated cargo molecules decreased, while the number of molecules released into the solution increased. The fraction ratio of released cargo molecules to the initially encapsulated ones in all micelles in the mixture is illustrated as a function of time in Figure 13 and Figure S9. The observed noise in the curves is a result of released cargo molecules or others from the solution being inserted into other micelles. In general, the cargo release rates mirror the depolymerization rates of copolymers. Specifically, at a constant concentration of trigger molecules and RP_T_ = 10^−4^, RP_B_ = 10^−3^, the release of cargo molecules is faster in linear A_30_B_30_ followed by A_30_(B_15_)_2_ and A_30_(B_10_)_3_ miktoarm copolymers. Conversely, for stoichiometric concentrations of trigger molecules with the end-cap beads, the release of cargo molecules is faster in A_30_(B_10_)_3_ followed by A_30_(B_15_)_2_ and linear A_30_B_30_ copolymers. This trend aligns with the decreasing hydrophobic content of B–type beads in micelles due to depolymerization and demicellization, allowing fewer hydrophobic cargo molecules to be accommodated. However, the fraction of cargo molecules released to the solution is not directly proportional to the fraction of depolymerized B–type beads. For a 0.60 degradation of hydrophobic beads, the fraction of cargo release is approximately 0.34, 0.39, and 0.43 for A_30_B_30_, A_30_(B_15_)_2_, and A_30_(B_10_)_3_, respectively, at both stoichiometric and constant concentrations of trigger molecules with RP_T_ = 10^−4^ and RP_B_ = 10^−3^. This is attributed to the existence of micelles with higher *N* in A_30_B_30_ than in the miktoarm copolymer mixtures, allowing them to accommodate a larger number of cargo molecules (Figure 9, Figure 10 and Figure 11). 

To quantitatively analyze the release of cargo molecules, the non-linear Korsmeyer–Peppas equation was employed [32,33]:

(4) $$\frac{{Μ}_{t}}{{Μ}_{\infty }}=kt^{n}$$

**Figure 14:**
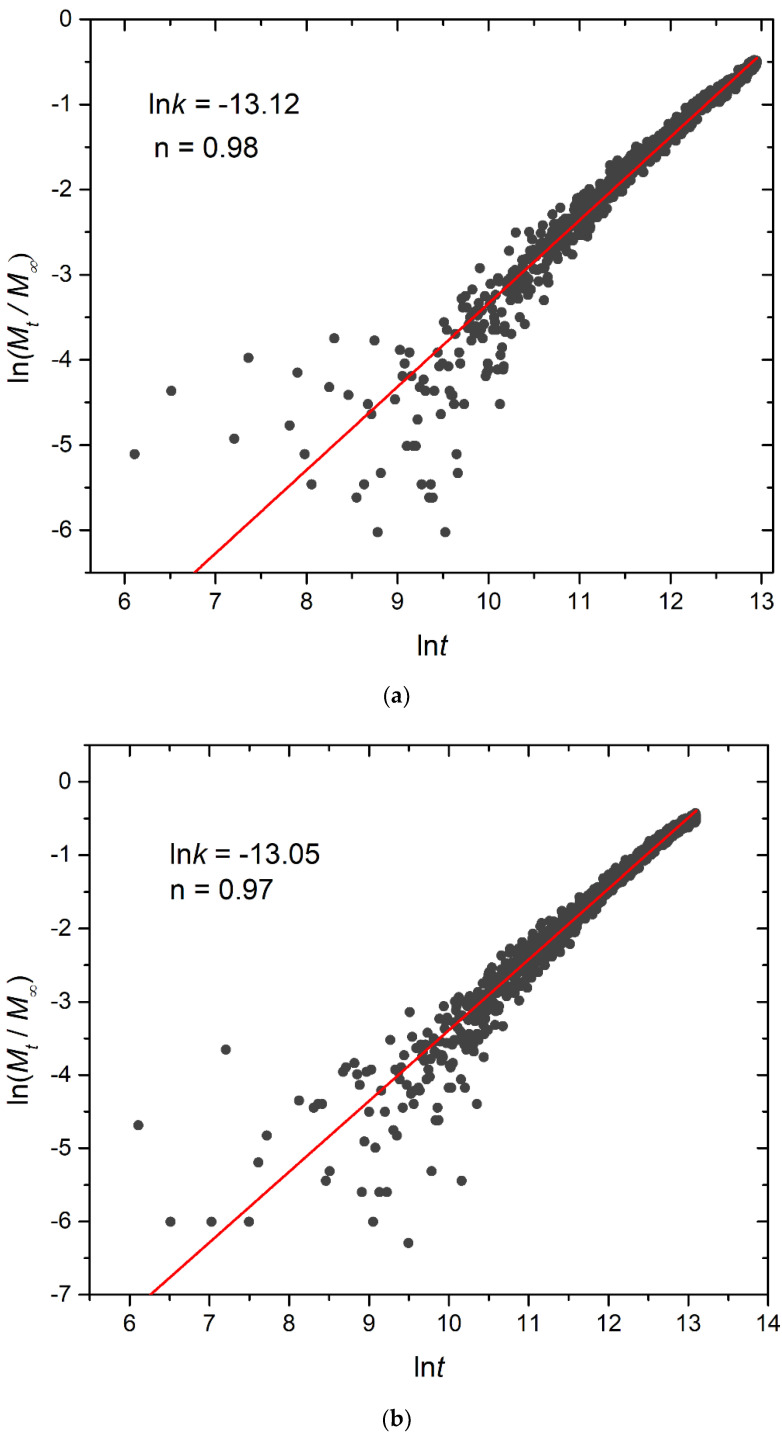

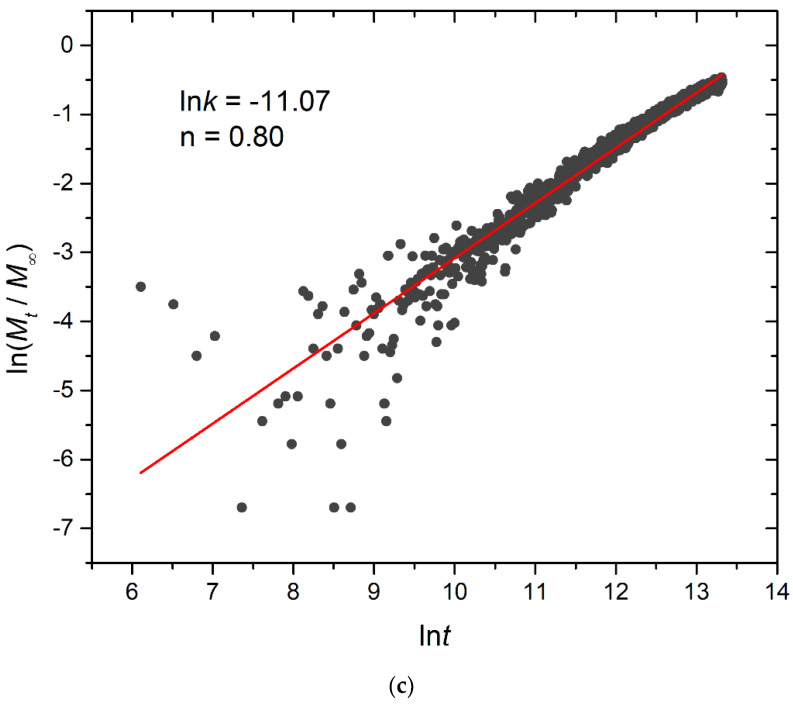
Plot depicting the fraction of released cargo molecules against time for (**a**) linear A_30_B_30_, (**b**) miktoarm A_30_(B_15_)_2_, and (**c**) miktoarm A_30_(B_10_)_3_ copolymers. Fitting lines, *k* and n, correspond to the Korsmeyer–Peppas equation. Trigger molecule concentration is maintained constant in all cases. RP_T_ = 10^−4^ and RP_B_ = 10^−3^.

In this equation, *M_t_*/*Μ*_∞_ signifies the fraction of released cargo molecules, *t* denotes the release time, *k* is the kinetic constant (with dimensions of τ^−1^), and n is the diffusional exponent for cargo release (dimensionless). The kinetic constant *k* primarily conveys information about the drug formulation’s characteristics, particularly those related to micelles, whereas *n* is related to the drug release mechanism. For 0.45 < n < 1, the release is non-Fickian, and both diffusion and micelle relaxation–corrosion contribute to the release of the mechanism [33]. The analysis focused on the initial 60% of the release curve. Plots for constant and stoichiometric trigger molecule concentrations (RP_T_ = 10^−4^ and RP_B_ = 10^−3^) are presented in Figure 14 and Figure S10, respectively. Notably, for constant trigger molecule concentrations, n values exhibit a decreasing trend from 0.98 to 0.97 to 0.80 as the number of hydrophobic blocks or branches increases, whereas the constant *k* values manifest the opposite trend. For stoichiometric mixtures, the variation of n is non-monotonic, with values ranging from 0.79 for A_30_B_30_, 1.18 for A_30_(B_15_)_2_, to 0.79 for A_30_(B_10_)_3_, respectively, and similarly for *k*. Consequently, our findings strongly suggest that the release of cargo from degradable micelles follows a non-Fickian pattern, where both diffusion and corrosion mechanisms contribute to the release process. This observation aligns with the consistent n values obtained in a previous study involving the cationic drug release from four-arm starblock copolymers, where the release behavior was dominated by chain relaxation–erosion induced by ion exchange that was dependent on pH [34]. A preview of the simulation box demonstrating depolymerization and cargo release is showcased in Videos S1 and S2.

Our results provide a qualitative description of the impact of copolymer architectures on demicellization and cargo release through head-to-tail depolymerization. However, for a quantitative analysis and comparison with experimental data, the MARTINI coarse-grained force field [35] proves to be more suitable. Therefore, such an investigation will be the focus of our future research in this field.

## 4. Conclusions

Our study aimed to investigate how the architecture of copolymers impacts demicellization and cargo release through head-to-tail depolymerization triggered by specific stimuli. Using comprehensive molecular dynamics simulations, we analyzed linear A_30_B_30_ copolymers and miktoarm star copolymers A_30_(B_15_)_2_ and A_30_(B_10_)_3_, differing in the number of hydrophobic arms and end-caps. The depolymerization process involved two steps: the rupture of bonds between hydrophobic end-caps and B–type beads initiated by T–type molecules (with predefined reaction probability RPT), followed by depolymerization in the absence of stimuli (with reaction probability RPB). Our investigation spanned various concentrations of trigger molecules relative to end-cap beads, exploring a range of RPT and RPB values.

Our findings demonstrated that micelle aggregation numbers (*N*_p_) were higher for linear copolymers and decreased with an increase in the number of arms in miktoarm copolymers. Although copolymer architecture did not affect the micelle loading capacity (LC), loaded micelles exhibited increased *N*_p_ and radius of gyration with higher numbers of hydrophobic branches. The depolymerization rate of hydrophobic beads was consistently faster in A_30_(B_10_)_3_ copolymers, followed by A_30_(B_15_)_2_ and A_30_B_30_ mixtures. This rate depended on the excess of trigger molecules and simultaneous head-to-tail depolymerization, with different copolymer compositions exhibiting varying trends. Cargo release rates generally mirrored depolymerization rates, but the fraction of released cargo was not directly proportional to depolymerized B–type beads. Utilizing the non-linear Korsmeyer–Peppas equation, our results indicated a non-Fickian release pattern where both diffusion and corrosion mechanisms contributed. These insights into copolymer behavior can guide the design of chains with more triggering groups, facilitating the development of delivery vehicles for diverse applications in biomedicine and environmental science.

## Data Availability

Data are contained within the article.

## Supplementary Materials

The following supporting information can be downloaded at: https://www.mdpi.com/article/10.3390/polym16081127/s1, Table S1: The number of encapsulated cargo molecules in micelles; Table S2: The preferential aggregation number (*N*_p_), the mean squared radius and gyration (<*R_g_^2^*>), and the shape asymmetry parameter (*κ*^2^) of micelles; Figure S1: Depolymerization fraction of end cap beads for constant trigger molecules concentration; Figure S2: Depolymerization fraction of end cap (ec) beads for 10 times the stoichiometric trigger molecules concentration; Figure S3: Mass distribution of micelles formed by linear A_30_B_30_ copolymers across various time points; Figure S4: Mass distribution of micelles formed by miktoarm A_30_(B_15_)_2_ copolymers across various time points; Figure S5: Mass distribution of micelles formed by miktoarm A_30_(B_10_)_3_ copolymers across various time points; Figure S6: Mass distribution of micelles formed by linear A_30_B_30_ copolymers across various time points (RP_T_ = 10^−2^ and RP_B_ = 10^−3^); Figure S7: Mass distribution of micelles formed by miktoarm A_30_(B_15_)_2_ copolymers across various time points (RP_T_ = 10^−2^ and RP_B_ = 10^−3^); Figure S8: Mass distribution of micelles formed by miktoarm A_30_(B_10_)_3_ copolymers across various time points (RP_T_ = 10^−2^ and RP_B_ = 10^−3^); Figure S9: Cargo molecules release fraction from A_30_B_30_, A_30_(B_15_)_2_, and A_30_(B_10_)_3_ copolymer mixtures plotted against time; Figure S10: Plot depicting the fraction of released cargo molecules against time for (a) linear A_30_B_30_, (b) miktoarm A_30_(B_15_)_2_, and (c) miktoarm A_30_(B_10_)_3_ copolymers; Video S1: A preview of simulation box showing the depolymerization of hydrophobic (red) beads in A_30_B_30_ copolymer mixtures; Video S2: A preview of simulation box showing the release of cargo molecules (green) in A_30_B_30_ copolymer mixtures.
