# Supplementary material for: Impact of Copolymer Architecture on Demicellization and Cargo Release via Head-to-Tail Depolymerization of Hydrophobic Blocks or Branches
Source: Polymers (Basel). 2024 Apr 17;16(8):1127. doi: 10.3390/polym16081127 (PMC11053811; doi:10.3390/polym16081127)
Supplement: Supplementary file 1 [file polymers-16-01127-s001.zip › polymers-2947668-supplementary.pdf]

Supporting Information

Impact of Copolymer Architecture on Demicellization and Cargo Release via Head-to-Tail Depolymerization of hydrophobic blocks or branches.

Christos Gioldasis[1], Apostolos Gkamas[1] and Costas Vlahos[1*]

[1] Chemistry Department, University of Ioannina, 45110 Ioannina, Greece

Table S1. The number of encapsulated cargo molecules in micelles formed from mixtures containing 1000 linear or miktoarm copolymer chains with 2000 or 4000 cargo molecules C_3. The different interaction parameters between hydrophobic C-C and B-C beads correspond to T^*=1.8,1.6, and 1.4. [Φ]=0.12.

	**Total C_3**	**encapsulated C_3**	**Ratio**
**$A_{30}B_{30}$, T^*_B = 1.8<br>T^*_C = 1.8, $T^*_{B\text{-}C}$ = 1.8**	2000	816	0.408
**$A_{30}B_{30}$, T^*_B = 1.8<br>T^*_C = 1.6, $T^*_{B\text{-}C}$ = 1.8**	2000	841	0.421
**$A_{30}B_{30}$, T^*_B = 1.8<br>T^*_C = 1.5, $T^*_{B\text{-}C}$ = 1.8**	2000	860	0.430
**$A_{30}B_{30}$, T^*_B = 1.8<br>T^*_C = 1.4, $T^*_{B\text{-}C}$ = 1.8**	2000	863	0.432
**$A_{30}B_{30}$, T^*_B = 1.8<br>T^*_C = 1.4, $T^*_{B\text{-}C}$ = 1.4**	2000	1662	0.831
**$A_{30}(B_{15})_2$, T^*_B = 1.8<br>T^*_C = 1.4, $T^*_{B\text{-}C}$ = 1.4**	2000	1651	0.826
**$A_{30}(B_{10})_3$, T^*_B = 1.8<br>T^*_C = 1.4, $T^*_{B\text{-}C}$ = 1.4**	2000	1625	0.812
**$A_{30}B_{30}$, T^*_B = 1.8<br>T^*_C = 1.4, $T^*_{B\text{-}C}$ = 1.4**	4000	3284	0.821

Table S2. The preferential aggregation number (N_p), the mean squared radius and gyration ($\langle R_g^2 \rangle$), and the shape asymmetry parameter (κ^2) of micelles formed by $A_{30}B_{30}$, $A_{30}(B_{15})_2$ and $A_{30}(B_{10})_3$ copolymers, and from their mixtures with 2000 C_3 cargo molecules.

	N_p	$\langle R_g^2 \rangle$	κ^2
**$A_{30}B_{30}$**	44	115.5 ± 0.2	0.0162 ± 0.0009
**$A_{30}B_{30}$ + C_3**	51	126.8 ± 0.3	0.0126 ± 0.0006
**$A_{30}(B_{15})_2$**	30	91.4 ± 0.2	0.031 ± 0.001
**$A_{30}(B_{15})_2$ + C_3**	39	108.4 ± 0.2	0.025 ± 0.001
**$A_{30}(B_{10})_3$**	23	82.1 ± 0.3	0.049 ± 0.002
**$A_{30}(B_{10})_3$ + C_3**	31	97.9 ± 0.4	0.041 ± 0.002

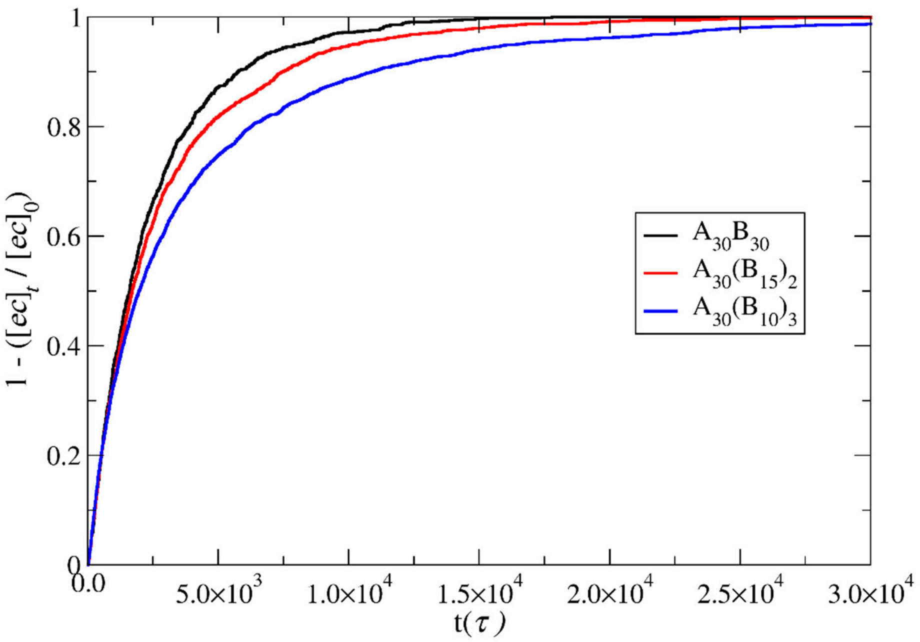

Figure S1 Depolymerization fraction of end cap beads of $A_{30}B_{30}$, $A_{30}(B_{15})_2$, and $A_{30}(B_{10})_3$ copolymers as a function of time for constant trigger molecules concentration. $RP_T=10^{-2}$, $RP_B=10^{-3}$. $[ec]_0$ is the initial end cap beads concentration, $[ec]_t$ is the end cap concentration.

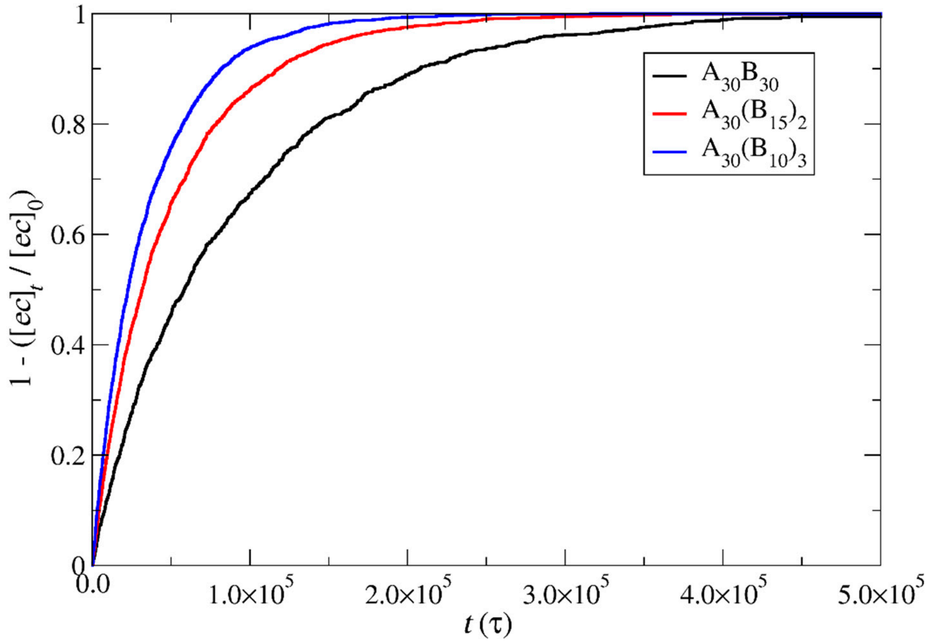

Figure S2 Depolymerization fraction of end cap (ec) beads of $A_{30}B_{30}$, $A_{30}(B_{15})_2$, and $A_{30}(B_{10})_3$ copolymers as a function of time for 10 times the stoichiometric trigger molecules

concentration. $RP_T=10^{-4}$, $RP_B=10^{-3}$. $[ec]_0$ is the initial end cap beads concentration, $[ec]_t$ is the end cap concentration.

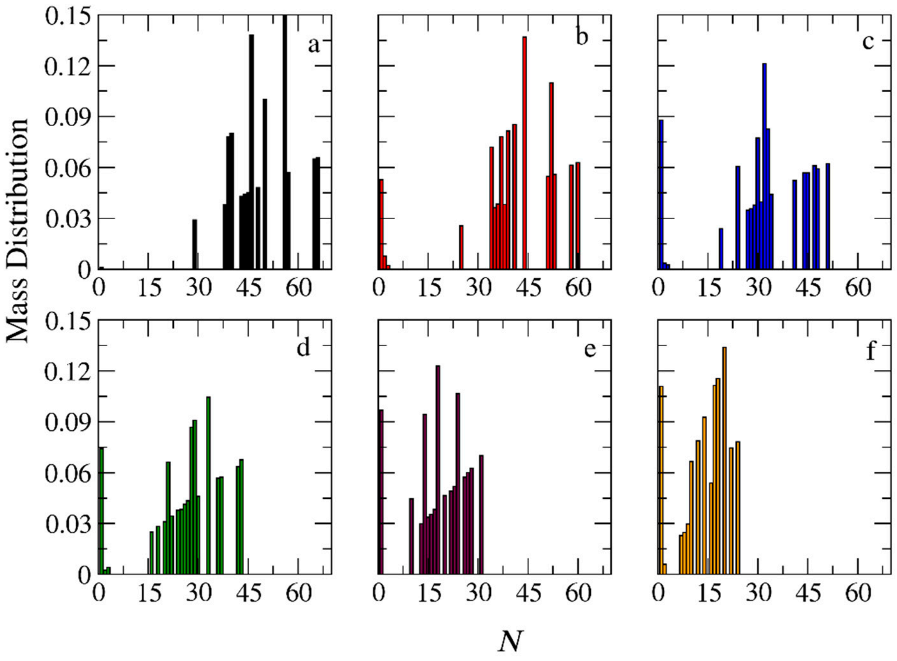

Figure S3 Mass distribution of micelles formed by linear $A_{30}B_{30}$ copolymers across various time points and depolymerization fractions of all hydrophobic beads: a) *t*=0, 0, b) *t*=9100τ, 0.17, c) *t*=13500τ, 0.32, d) *t*=18000τ, 0.44, e) *t*= 27000τ, 0.61, f) *t*= 36500τ, 0.74. The trigger molecule concentration is maintained constant in all cases. $RP_T=RP_B=10^{-3}$.

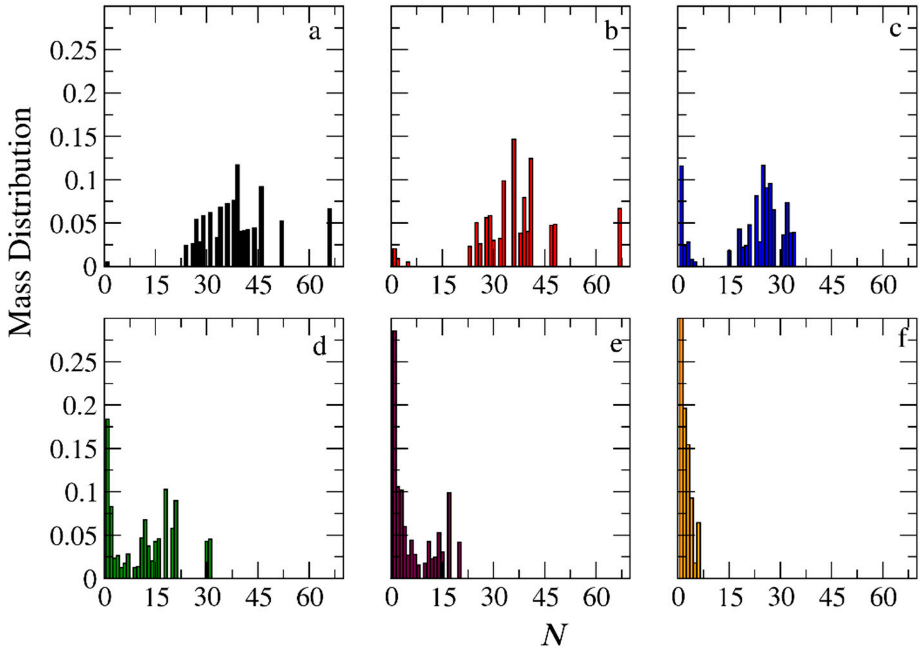

Figure S4 Mass distribution of micelles formed by miktoarm $A_{30}(B_{15})_2$ copolymers across various time points and depolymerization fractions of all hydrophobic beads: a) t=0, 0, b) t=4500τ, 0.10, c) t=9000τ, 0.24, d) t=18000τ, 0.45, e) t= 27000τ, 0.59, f) t= 45000τ, 0.76. The trigger molecule concentration is maintained constant in all cases. $RP_T = RP_B = 10^{-3}$.

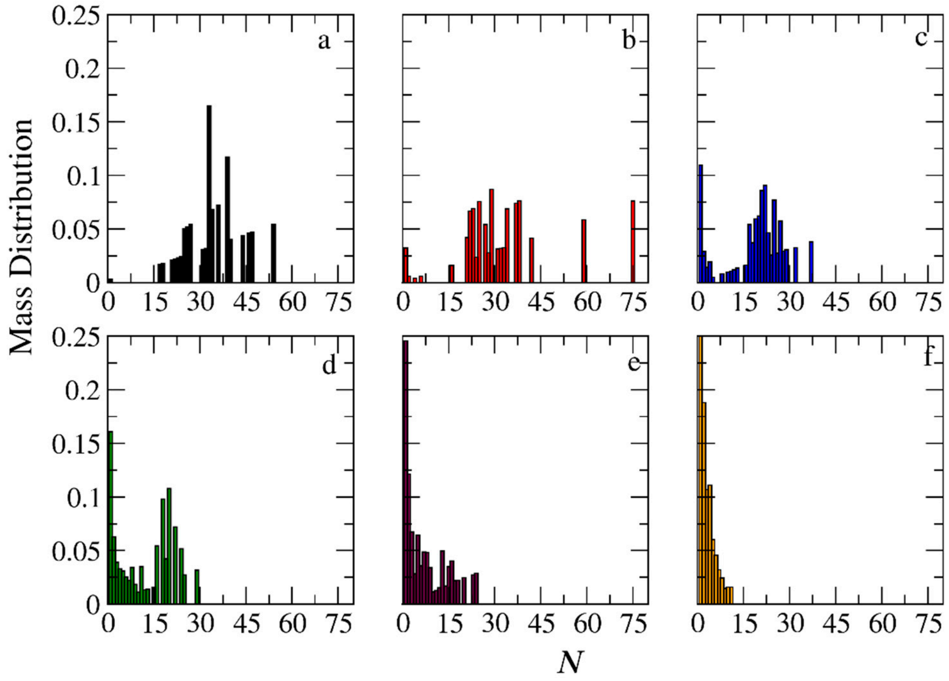

Figure S5 Mass distribution of micelles formed by miktoarm $A_{30}(B_{10})_3$ copolymers across various time points and depolymerization fractions of all hydrophobic beads: a) t=0, 0, b)

t=4500τ, 0.11, c) *t*=13500τ, 0.34, d) *t*=22500τ, 0.49, e) *t*= 36000τ, 0.63, f) *t*= 63000τ, 0.76. The trigger molecule concentration is maintained constant in all cases. $RP_T=RP_B=10^{-3}$

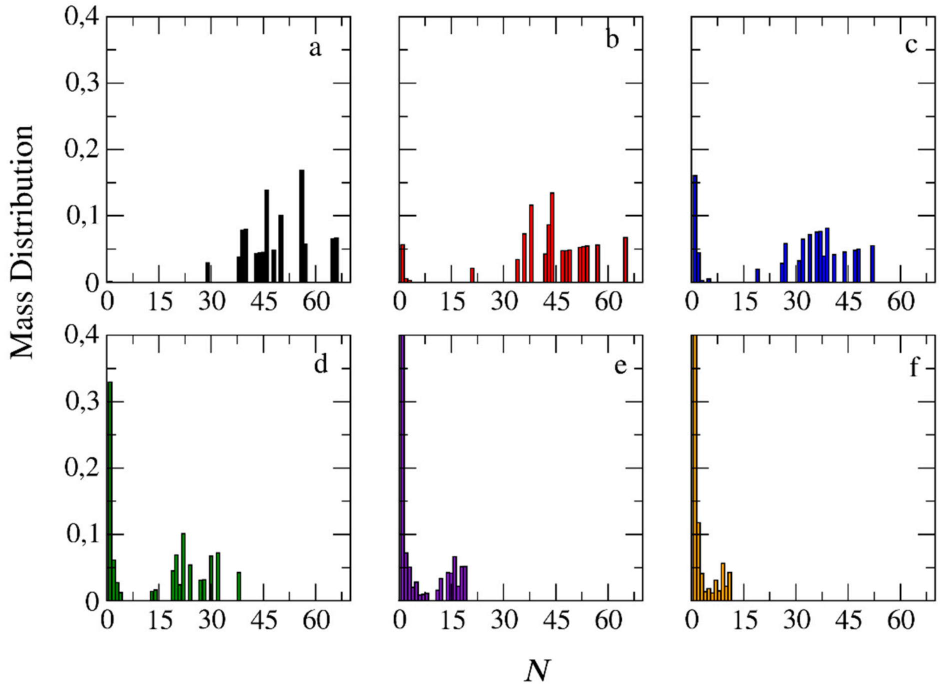

Figure S6 Mass distribution of micelles formed by linear $A_{30}B_{30}$ copolymers across various time points and depolymerization fractions of all hydrophobic beads: a) *t*=0, 0, b) *t*=4500τ, 0.27, c) *t*=6000τ, 0.41, d) *t*=7500τ, 0.55, e) *t*= 9000τ, 0.68, f) *t*= 10500τ, 0.80. The trigger molecule concentration is maintained constant in all cases. $RP_T=10^{-2}$ and $RP_B=10^{-3}$.

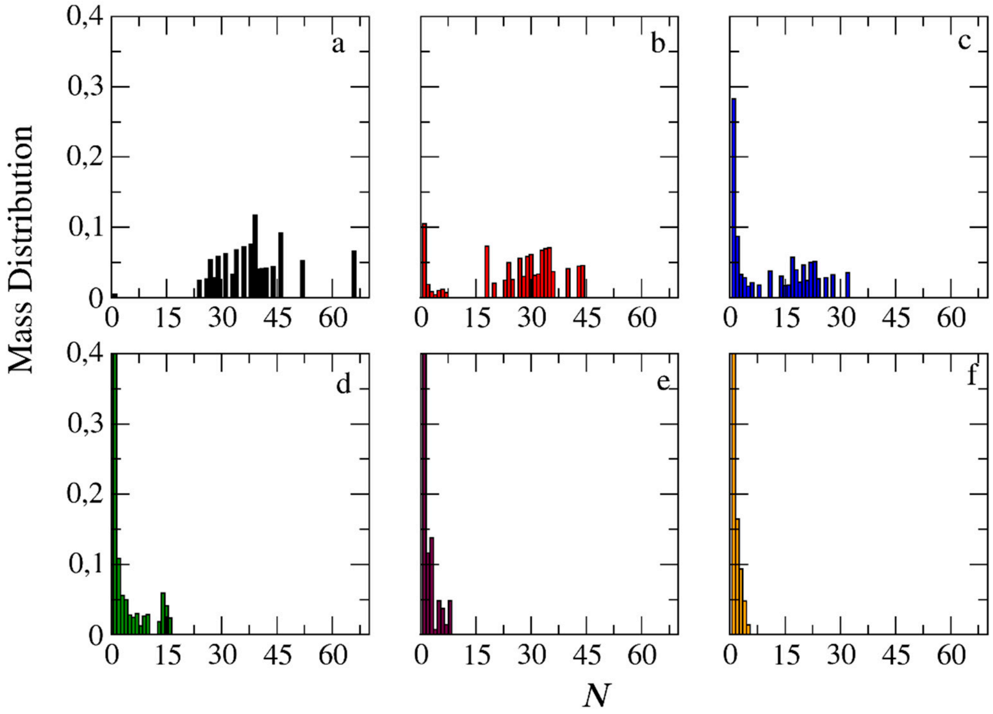

Figure S7 Mass distribution of micelles formed by miktoarm $A_{30}(B_{15})_2$ copolymers across various time points and depolymerization fractions of all hydrophobic beads: a) *t*=0, 0, b) *t*=3000τ, 0.30, c) *t*=4000τ, 0.45, d) *t*=6000τ, 0.67, e) *t*= 7500τ, 0.78, f) *t*= 9000τ, 0.86. The trigger molecule concentration is maintained constant in all cases. $RP_T=10^{-2}$ and $RP_B=10^{-3}$.

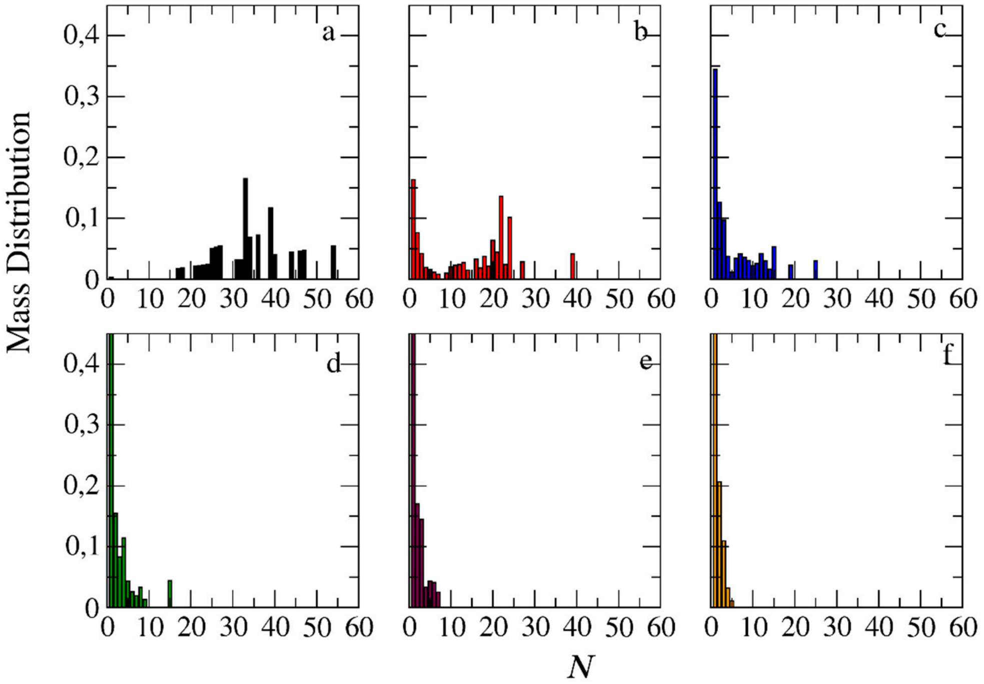

Figure S8 Mass distribution of micelles formed by miktoarm $A_{30}(B_{10})_3$ copolymers across various time points and depolymerization fractions of all hydrophobic beads: a) *t*=0, 0, b) *t*=3000τ, 0.37, c) *t*=4500τ, 0.56, d) *t*=6000τ, 0.68, e) *t*= 7500τ, 0.74, f) *t*= 9000τ, 0.80. The trigger molecule concentration is maintained constant in all cases. $RP_T=10^{-2}$ and $RP_B=10^{-3}$.

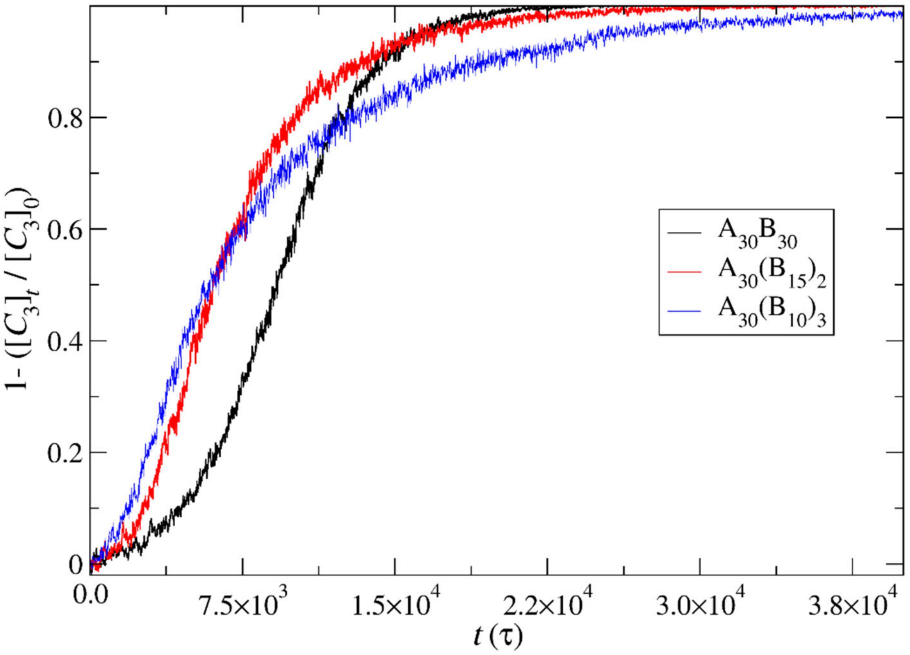

a

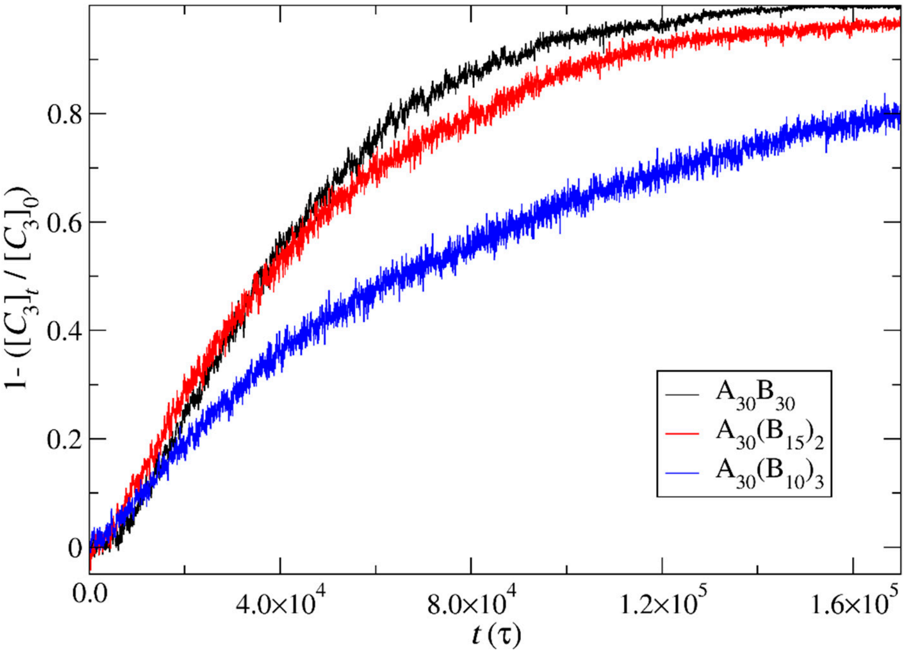

b

Figure S9 Cargo molecules release fraction from $A_{30}B_{30}$, $A_{30}(B_{15})_2$, and $A_{30}(B_{10})_3$ copolymer mixtures plotted against time for constant trigger molecule concentration. a) $RP_T=10^{-2}$, $RP_B=10^{-3}$ and b) $RP_T=10^{-3}$ and $RP_B=10^{-3}$. $[C_3]_0$ is the initial cargo molecules concentration, $[C_3]_t$ is the cargo molecules concentration.

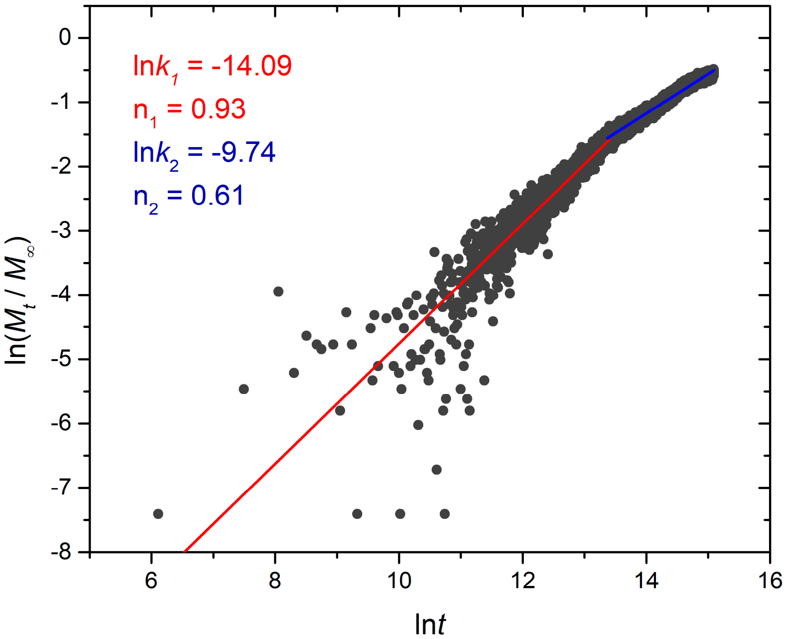

a

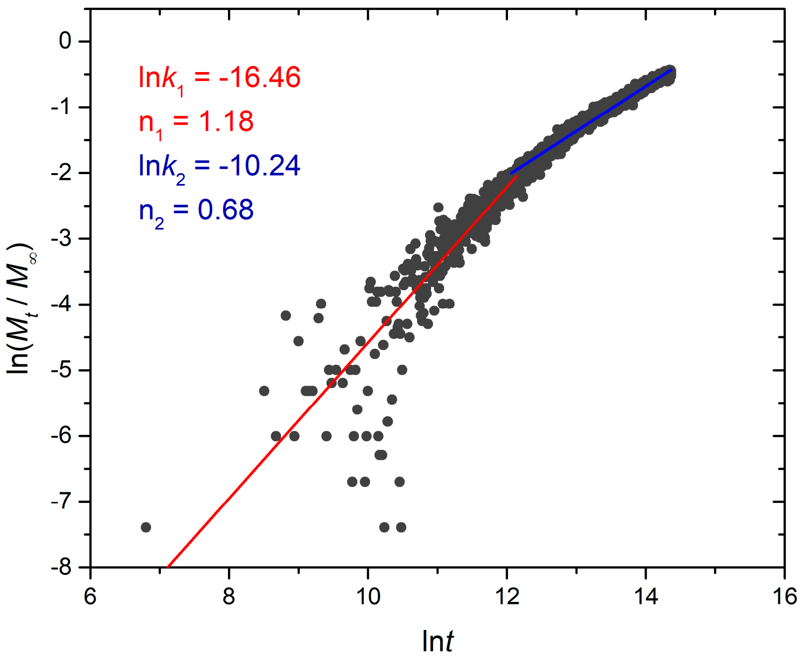

b

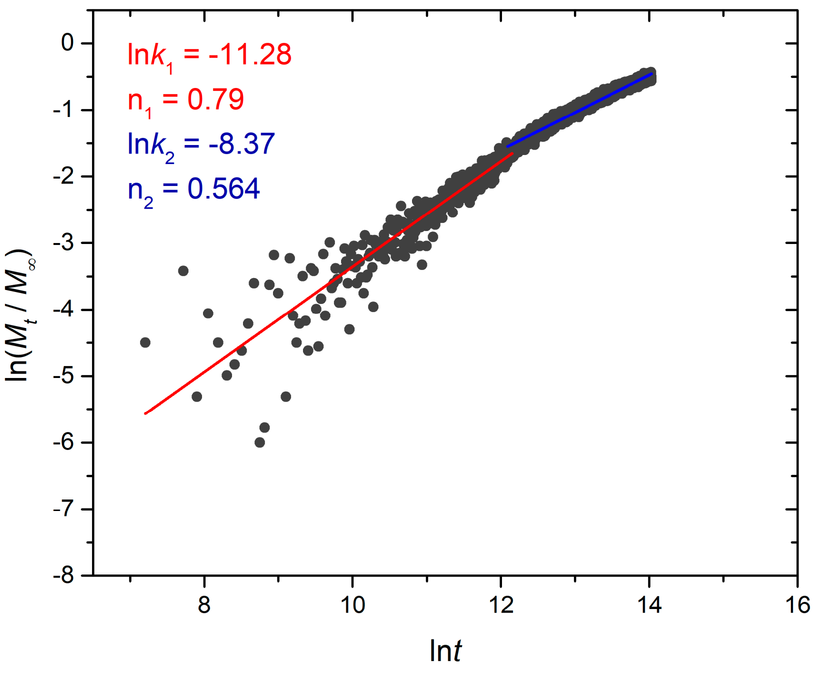

c

Figure S10 Plot depicting the fraction of released cargo molecules against time for a) linear $A_{30}B_{30}$, b) miktoarm $A_{30}(B_{15})_2$, and c) miktoarm $A_{30}(B_{10})_3$ copolymers. Fitting lines, k and n

correspond to the Korsmeyer-Peppas equation. Trigger molecule concentration is maintained stoichiometric to end cap beads in all cases. $RP_T=10^{-4}$ and $RP_B=10^{-3}$.

Radius of Gyration

The radii of gyration used to describe the dimensions of a polymeric micelle $< R_g^2 >_{micelle}$ and the micelle's core $< R_g^2 >_{core}$ are defined as:

$$< R_g^2 >_{micelle} = \frac{1}{M}\sum_{i=1}^{M}(\boldsymbol{r}_i - \boldsymbol{r}_{CM})^2 \qquad \text{(S1)}$$

$$< R_g^2 >_{core} = \frac{1}{M_c}\sum_{i=1}^{M_c}(\boldsymbol{r}_i - \boldsymbol{r}_{coreCM})^2 \qquad \text{(S2)}$$

where, M_C represents the number of B-type beads forming the micelle core, and M denotes the total number of beads (both A-type and B-type) forming the micelle. $\boldsymbol{r}$, $\boldsymbol{r}_{CM}$, and $\boldsymbol{r}_{coreCM}$ denote the position vectors of i bead, the center of mass of B-type beads, and the center of mass of all beads forming the micelle, respectively.

Shape anisotropy parameter.

The shape anisotropy κ^2 is defined as(Theodorou & Suter, 1985):

$$\kappa^2 = 1 - 3\frac{<I_2>}{<{I_1}^2>}, \qquad \text{(S3)}$$

where I_1 and I_2 are the first and second invariants of the radius of gyration tensor. $\kappa^2 = 0$ corresponds to a perfect sphere while $\kappa^2 = 1$ to a perfect rod. The relationship between the packing parameter and the shape anisotropy parameter in polymers is not a direct one, but they are both influenced by molecular structure and interactions. In some cases, molecules with high shape anisotropy (high κ^2) may have difficulty packing efficiently, especially in self-assembly processes where spherical structures are favored. Conversely, molecules with low shape

anisotropy (low κ^2) may have a higher tendency to pack efficiently. However, this relationship can vary depending on the specific molecular interactions and the overall system conditions.

SV1.mp4

Video S1. A preview of simulation box showing the depolymerization of hydrophobic (red) beads in $A_{30}B_{30}$ copolymer mixtures for constant trigger molecule concentration is presented ($RP_T=10^{-4}$, $RP_B=10^{-3}$). Cargo molecules are shown in green. For clarity all other types of beads are hidden.

SV2.mp4

Video S2. A preview of simulation box showing the release of cargo molecules (green) in $A_{30}B_{30}$ copolymer mixtures for constant trigger molecule concentration is presented ($RP_T=10^{-4}$, $RP_B=10^{-3}$). For clarity all other types of beads are hidden.
